# Comparison of Ethanolic and Aqueous *Populus balsamifera* L. Bud Extracts by Different Extraction Methods: Chemical Composition, Antioxidant and Antibacterial Activities

**DOI:** 10.3390/ph14101018

**Published:** 2021-10-02

**Authors:** Monika Stanciauskaite, Mindaugas Marksa, Lina Babickaite, Daiva Majiene, Kristina Ramanauskiene

**Affiliations:** 1 Department of Clinical Pharmacy, Faculty of Pharmacy, Lithuanian University of Health Sciences, Sukileliai Avenue 13, LT-50161 Kaunas, Lithuania; Kristina.ramanauskiene@lsmuni.lt; 2 Department Analytical & Toxicological Chemistry, Faculty of Pharmacy, Lithuanian University of Health Sciences, Sukileliai Avenue 13, LT-50161 Kaunas, Lithuania; mindaugas.marksa@lsmu.lt; 3 Dr. L. Kriaučeliūnas Small Animal Clinic, Veterinary Academy, Lithuanian University of Health Sciences, Tilzes Str. 18, LT-47181 Kaunas, Lithuania; lina.babickaite@lsmuni.lt; 4 Laboratory of Biochemistry, Neuroscience Institute, Lithuanian University of Health Sciences, Eiveniu Str. 4, LT-50162 Kaunas, Lithuania; daiva.majiene@lsmuni.lt; 5 Department of Drug Technology and Social Pharmacy, Lithuanian University of Health Sciences, Sukileliu Str. 13, LT-50162 Kaunas, Lithuania

**Keywords:** balsam poplar buds, extraction, phenolic compounds, flavonoids, biological activity

## Abstract

The balsam poplar (*Populus balsamifera* L.) buds that grow in Lithuania are a polyphenol-rich plant material with a chemical composition close to that of propolis. In order to potentially adapt the extracts of this plant’s raw material for therapeutic purposes, it is important to carry out detailed studies on the chemical composition and biological activity of balsam poplar buds. An important step is to evaluate the yield of polyphenols by different extraction methods and using different solvents. According to our research, extracts of balsam poplar buds collected in Lithuania are dominated by *p*-coumaric (496.9–13,291.2 µg/g), cinnamic acid (32.9–11,788.5 µg/g), pinobanksin (34.9–1775.5 µg/g) and salicin (215.3–1190.7 µg/g). The antioxidant activity of poplar buds was evaluated by the ABTS (2,2-azino-bis(3-ethylbenzothiazoline-6-sulfonic acid), DPPH (2,2-diphenyl-1-picrylhydrazyl) and FRAP (ferric-reducing antioxidant power) methods, all extracts showed antioxidant activity and the obtained results correlated with the obtained amounts of total phenolic compounds in the extracts (ABTS r = 0.974; DPPH r = 0.986; FRAP r = 0.955, *p* < 0.01). Studies of antimicrobial activity have shown that ethanolic extracts have an antimicrobal activity effect against *Staphylococcus aureus*, *Enterococcus faecalis* and *Escherichia coli*. The extracts showed a better antimicrobal activity against gram-positive bacteria.

## 1. Introduction

In traditional and ancient medicine, medicinal plants from prevailing geographical areas were used. Medicinal plants were used for self-treatment in order to improve chronic ailments and inflammatory conditions [1]. Phytotherapy is based on the action of active compounds [2]. It is known since ancient times that phenolic acids and flavonoids have anti-inflammatory, antioxidant and antimicrobial effects, but more and more attention is given to studies on the anti-tumor action of polyphenols [3]. These biologically active compounds are important not only for the elimination of inflammatory reactions in the organism, but also because they serve as a wonderful preventive aid. The use of raw plant material and bee products for cosmetic and pharmaceutical purposes depends on cumulative polyphenols and their quantity. Bee products have been greatly valued since ancient times, especially propolis, as it is rich in phenolic acids and flavonoids [4,5]. Propolis was widely used for wound and skin care, for the treatment of bacterial and viral upper respiratory tract infections, for immune system strengthening and for the treatment of gastric problems [6,7]. Propolis is widely used and valued in different continents, but the obtainment of its raw material is a complicated and lengthy process, and not all people use products of animal origin. Due to these reasons, more and more attention is being paid to plant raw material, whose chemical composition of polyphenols is very close to that of propolis [8]. Scientists are increasingly studying the chemical composition and biological activity of plants from which propolis is collected, and the available results show that the chemical composition of propolis depends on the dominant plants in the collection area [9].

More attention is being paid to the *Populus* species—one of the main source of resins for bees [10]. These species (*Populus* spp., Salicaceae) are very adaptable and regenerate easily. Traditional medicine greatly values the trees of the *Populus* family, which are widely spread in North America, Europe and Asia [11]. Some scientific sources note that the use of poplar buds was first described in John Gerard’s book in 1597, as an ointment for inflammation [12]. Flavonoids, minerals, resins, glycosides, organic acids and waxes can be detected in balsam poplar buds. The main ones are phenolic compounds, which determine the antioxidant, anti-inflammatory and antimicrobial properties of this plant’s raw material [12,13,14]. Research has shown the antifungal activity of poplar buds against a *Penicillinium italicum* mold [15]. Castaldo and Capasso (2002) showed that propolis extracts displayed antimicrobial activity, mainly against gram-positive bacteria (*Staphylococcus* spp. and *Streptococcus* spp.) [16]. According to the similarities in the chemical composition of propolis and poplar buds, it is important to study the antimicrobial activity of the poplar buds growing in Lithuania. The anti-inflammatory and antioxidant effects of poplar buds have been studied by Chinese researchers using DPPH and ABTS free radical scavenging methods [17]. Simard et al. reported the isolation of 12 new flavan derivatives from *P. balsamifera* buds which have been isolated as six pairs of enantiomers. Other scientists investigated the antibacterial activity and the cytotoxicity of these compounds against *S. aureus* and human skin fibroblast cells [18].

In Lithuania, poplar buds are only used for the digestive system in traditional herbal medicine. The initial research on the chemical composition of balsam poplar buds was carried out in Lithuania and determined that it consisted mainly of *p*-coumaric and cinnamic acids [19]. The researchers confirmed that the extracts of balsamic poplar buds collected in Lithuania contain predominant phenolic compounds that coincide with the predominant Lithuanian propolis phenolic compounds [20].

It is important to produce liquid extracts from balsam poplar buds in order to target them in the fields of cosmetics, pharmaceuticals and food industry. An important step in the production of extracts is the choice of solvent and the conditions of the extraction process. The most popular technique for producing plant liquid extracts is the extraction with alcoholic solvents such as ethanol or methanol [21,22]. The extraction of plant material with methanol is a method that is simple and effective. However, it has drawbacks, such as restrictions on the use in the cosmetics and pharmaceutical industries. For example, ethanol and methanol extracts in medicine are not suitable for some ophthalmological diseases, pediatric diseases, otorhinolaryngological diseases or alcohol intolerance, requiring an alternative preparation of propolis extracts [23]. Therefore, it is important to produce non-ethanolic plant extracts based on efficient non-ethanolic extraction technologies. Quite often, purified water is used as a solvent in the production of non-ethanolic extracts [23]. It is safe and convenient to use water for the production of extracts, but mixtures of water and ethanol are used to extract more biologically active compounds.

There is not much information in the scientific literature about the production of balsam poplar buds’ aqueous extracts. Biologically active substances are usually sparingly soluble in water, and thus the content of active compounds is lower than in ethanol extracts [24]. It is important to find effective co-solvents that increase the water solubility of these substances. Another important step in the production of extracts is the selection of conditions for the extraction process [24,25]. The heating and cooling times are important when producing plant water extracts by the hot extraction method [25]. Infusions usually heat in 15 min and cool down in 45 min. Decoctions heat in 30 min and cool down in 15 min [26,27]. This extraction method is cheap, as it requires no special equipment, but in terms of time it is a rather long process. Due to the long-lasting diffusion of compounds, maceration is less and less used in various studies [28]. In order to reduce the extraction time, ultrasonic extraction is increasingly used for the separation of active compounds from plant raw materials. The application of ultrasounds in extraction is simple, economical and more efficient than other traditional extraction methods due to the high extraction yield and short extraction time [28,29].

Due to the fact that poplar buds have a wide biological impact and can potentially be targeted in the production of dermatological preparations, ophthalmological preparations and oral pharmaceutical forms, it is important to produce liquid extracts for their production using aqueous and ethanolic solvents. The aim of this study is to prepare liquid aqueous and ethanolic extracts of *Populus balsamifera* L. buds, to investigate the differences in their chemical composition depending on the extraction conditions and to determine their antioxidant and antimicrobial activity in vitro.

## 2. Results

### 2.1. Total Phenolic Compounds and Total Flavonoids

Having conducted the analysis of the phenolic compounds, and according to the results obtained, it became clear that the ethanol extracts had more phenolic compounds in comparison with purified water (Table 1). A Student’s t-test revealed that the difference in quantity of phenolic compounds in ethanol extracts and purified water was statistically significant. When comparing ultrasound-assisted samples, 99.62 mg CAE/g FW more phenols (t(4) = 18.77, *p* < 0.0001) and 43.05 mg RE/g FW more flavonoids (t(4) = 27.33, *p* = 0.001) were extracted from fresh balsam poplar samples in ethanol. In the dried balsam poplar buds, the concentrations were higher for phenols (difference of 1.11 mg CAE/g DW, t(4) = 1.31, *p* < 0.001) and flavonoids (difference of 3.53 mg RE/g DW, t(4) = 2.62, *p* < 0.001) as well. The macerated fresh poplar bud samples produced 84.7 mg CAE/g FW more phenols in ethanol samples compared to aqueous samples (t(4) = 13.79, *p* = 0.003) and 3.80 mg RE/g FW more flavonoids (t(4) = 3.15, *p* < 0.001), while the macerated dried poplar bud samples produced 115.35 mg CAE/g DW more phenols (t(4) = 15.20, *p* < 0.001) and 29.49 mg RE/g DW more flavonoids (t(4) = 24.43, *p* = 0.001).

When assessing the influence of the hot extraction method on the extraction of active compounds from the plant raw material, it was determined that decoctions had a better output of phenolic compounds than infusions: 1.75 mg CAE/g FW of phenols (t(4) = −12.61, *p* < 0.001), and 0.50 mg RE/g FW flavonoids (t(4) = −4.17, *p* = 0.02) were extracted from fresh poplar buds, and 2.44 mg CAE/g DW more phenols (t(4) = −11.21, *p* < 0.001) and 2.40 mg RE/g DW more flavonoids (t(4) = −10.75, *p* = 0.001) were extracted from dried poplar buds.

Maceration and ultrasound-assisted extraction improves the output of water extracts in comparison with infusions and decoctions. In view of the research results, there was no difference when using maceration or ultrasound-assisted extraction (a difference of 6.58 mg CAE/g FW phenols, A1U and A1M, t(4) = −2.14, *p* = 0.11). However, 17.05 mg CAE/g DW more phenols were extracted from the dried samples (B1U and B1M, t(4) = −3.79, *p* = 0.02). In ethanol samples it was determined that when maceration and ultrasound-assisted therapy was used, there was a marginal statistically significant difference between the total amount of phenolic compounds in produced ethanolic extracts in the fresh poplar buds (A2U and A2M, difference of 21.5 mg CAE/g FW phenols, t(4) = 2.83, *p* = 0.049) as well as the dried buds (a difference of 13.1 mg CAE/g DW phenols, B2U and B2M, t(4) = 1.25, *p* = 0.28).

Using infusions, 2.26 mg CAE/g more phenolic compounds were extracted from the dried buds compared to the fresh buds (t(4) = −14.78, *p* < 0.001). Using decoctions, 2.95 mg CAE/g more phenols were extracted (t(4) = −14.22, *p* < 0.001). When comparing the raw and dried plant material in the ethanolic solution, macerating the dried plant material produced 19.47 mg CAE/g more phenols (t(4) = 5.27, *p* = 0.01), and ultrasound-assistance produced 29.95 mg CAE/g more phenols (t(4) = 7.5, *p* = 0.002). The total output of flavonoids was much higher from the ethanol extracts than the aqueous extracts.

### 2.2. HPLC Analysis

To evaluate the predominant phenolic compounds in the balsam poplar, a high-performance liquid chromatography analysis was performed for all aqueous and ethanol extracts.

The HPLC analysis showed that in the aqueous and ethanolic extracts of balsam poplar buds the predominant phenolic acids were *p*-coumaric, caffeic and cinnamic acids (Table 2). The highest amount of *p*-coumaric acid was noticed in all extracts. According to a Student’s t-test, a statistically significant higher amount of *p*-coumaric acid was noticed in all the aqueous extracts (B1I, B1D, B1M, B1U) which were produced from dried plant material, compared to the extracts (A1I, A1D, A1M, A1U) which were produced from fresh poplar buds (F(1) = 1743.50, *p* < 0.001). In the dry plant material extracts, a higher amount of cinnamic acid was determined in comparison with the extracts of fresh plant material (F(1) = 1682.93, *p* < 0.001).

In the ethanolic extracts, a higher amount of flavonoids was determined compared to the aqueous extracts. In the aqueous extracts, only a small amount of flavonoids was noticed. The predominant flavonoids in the ethanolic balsam poplar bud extracts were pinocembrin and pinobanksin. A statistically significant higher (F(1) = 217.58, *p* < 0.001) amount of pinocembrin was found in the fresh ethanolic balsam poplar bud extracts in comparison with the dried ethanolic extracts.

A higher amount of salicin was noticed in the aqueous extracts compared to the ethanolic extracts. On the basis of the obtained results, it was noticed that the biggest amount of salicin was extracted from the fresh plant material while making infusions and decoctions.

**Figure 1:**
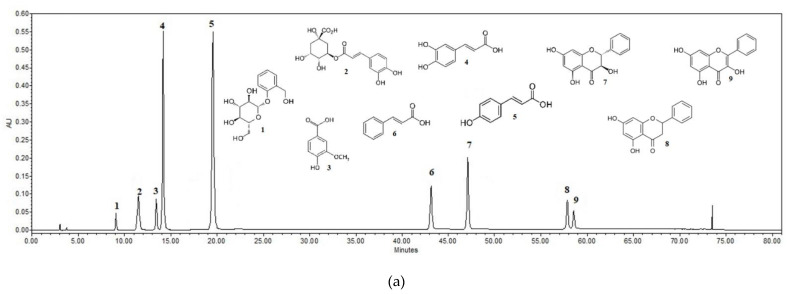

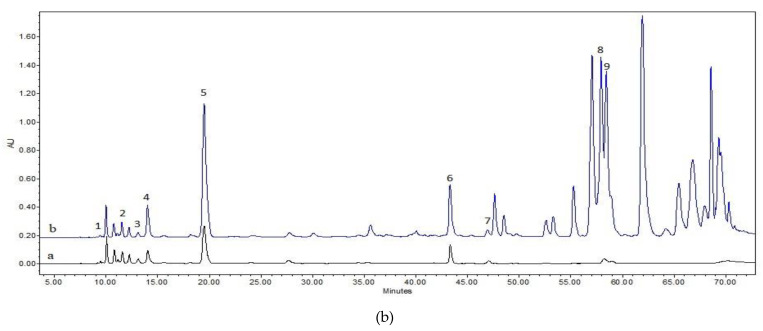
HPLC chromatograms of the standard compounds and identified compounds in the extracts. Upper Standard compounds’ chromatogram: 1. salicin (RT = 9.042; R^2^ = 0.9999), 2. chlorogenic acid (RT = 11.538; R^2^ = 0.9999), 3. vanillic acid (RT = 13.456; R^2^ = 0.9999), 4. caffeic acid (RT = 14.084; R^2^ = 0.9999), 5. *p*-coumaric acid (RT = 19.669; R^2^ = 0.9999), 6. cinnamic acid (RT = 43.246; R^2^ = 0.9999), 7. pinobanksin (RT = 47.158, R^2^ = 0.9999), 8. pinocembrin (RT = 57.967; R^2^ = 0.9998), 9. galangin (RT = 58.636; R^2^ = 0.9998). The lower chromatogram shows the identified compounds of the aqueous dried balsam poplar bud extract B1U (**a**) and the ethanolic dried balsam poplar bud extract B2U (**b**) extracted by ultrasound: 1. salicin, 2. chlorogenic acid, 3. vanillic acid, 4. caffeic acid, 5. *p*-coumaric acid, 6. cinnamic acid, 7. pinobanksin, 8. pinocembrin, 9. galangin.

The results of the HPLC analysis showed that a higher amount of biologically active compounds was determined in the fresh ethanolic balsam poplar bud extracts in comparison with the aqueous extracts when extracted via maceration (a difference of 6273.5 µg/g, t(4) = 62.51, *p* < 0.001) as well as ultrasound-assistance (a difference of 9659.4 µg/g, t(4) = 53.35, *p* < 0.001). A higher amount of phenolic acids was noticed in the ethanolic extracts from dried raw material than in the extracts from fresh raw material. A statistically significant higher amount of phenolic acids was noticed in the ethanolic extracts in comparions with the aqueous extracts when extracted via maceration (a difference of 2247.6 µg/g, t(4) = 29.86, *p* < 0.001) as well as ultrasound-assistance (a difference of 5384.7 µg/g, t(4) = 33.04, *p* < 0.001). The characteristic chromatograms of active compounds’ standards and the extracts of ethanol and aqueous poplar buds are given in Figure 1.

### 2.3. Antioxidant Activity

**Figure 2:**
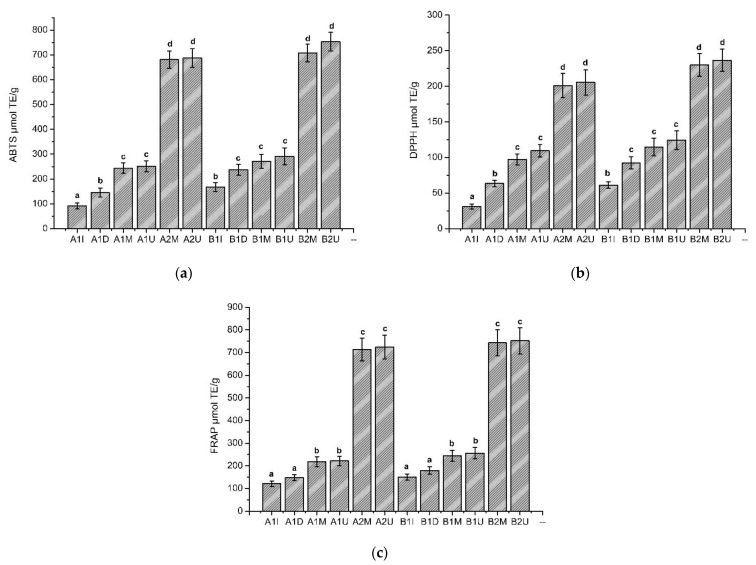
The antioxidant activity of balsam poplar bud extracts samples in vitro by (**a**) ABTS, (**b**) DPPH, (**c**) FRAP methods (fresh and dried balsam poplar buds were used for extraction). The extracts made from fresh plant material are marked with the letter A, the extracts made from dried plant material are marked with the letter B. The extracts prepared by different methods are marked accordingly: I-infusions, D-decoctions, M-maceration, U-ultrasound. Different solvents were used for the extraction, which are denoted respectively: 1-purified water, 2-70% ethanol (*v/v*)). The results are expressed as µmol of the trolox equivalent per g (µmol TE/g). The different letters in superscript (a–d) indicate a statistical difference between the antioxidant activity of the extracts by a Student’s *t*-test (*p* < 0.05).

Three methods have been used to measure the antioxidant activity of balsam poplar bud extracts—the ABTS, DPPH and FRAP radical scavenging assays (Figure 2)—and compared according to a Student’s t-test. In the antioxidant activity study, all the extracts showed antioxidant activity in vitro. The ABTS free radical scavenging capacity of various balsam poplar bud extracts samples is shown in Figure 2a. As can be seen in Figure 2, the ethanolic balsam poplar bud extracts have a statistically significant (F(1) = 639.86, *p* < 0.001) higher antioxidant activity using the ABTS method compared to the aqueous extracts. The DPPH free radical scavenging capacity of the balsam poplar bud extracts samples is shown in Figure 2b and a statistically significant (F(1) = 160.22, *p* < 0.001) stronger antioxidant activity of the ethanolic extracts compared to the aqueous balsam poplar bud extracts was also observed. Using the FRAP reductive activity method, the results in Figure 2c correlate with the results obtained with ABTS and DPPH, and a stronger reductive activity is observed in the ethanolic balsam poplar bud extracts (F(1) = 482.84, *p* < 0.001).

### 2.4. Antimicrobal Activity

The results of the antimicrobial study showed (Table 3) that the aqueous balm poplar extracts have a weak effect on the gram-positive bacteria *S. aureus* and *E. faecalis* and do not act against the gram-negative bacteria *E. coli* and *P. aeruginosa*. When performing a two-way ANOVA test, a statistically significant effect of the extracts on gram-positive bacteria was observed compared to gram-negative ones (F(4) = 467.71, *p* < 0.001). The extracts of dried raw material had a statistically significant stronger antimicrobial activity compared to the ethanolic extracts of fresh raw material (F(3) = 294.10, *p* < 0.001). For the wild-type *S. aureus*, dried macerated samples showed a stronger inhibition compared to fresh samples (an 8.0 mm [6.30; 9.70] difference) as well as ultrasound-assisted samples (a 10.0 mm [8.30; 11.70] difference). The same was true for gram-negative bacteria: for the wild-type *E. faecalis*, the dried macerated samples (a 4.0 mm [2.72; 5.28] change) as well as the dried ultrasound assisted samples (a 5.6 mm [4.32; 6.89] change) showed larger inhibition compared to the fresh samples. As a negative control of balsam poplar buds solvent, 70% ethanol (*v/v*) did not show a significant antibacterial activity on the studied strains. These results suggest that the antibacterial effect on the selected microorganisms was due to the constituents of the balsamic poplar bud extracts.

### 2.5. Correlation

A significant positive relationship between total phenolic compounds and antioxidant activity was observed (Table 4). A strong correlation was observed between the total amount of phenolic compounds and antioxidant activity (FRAP r = 0.955; ABTS r = 0.974; DPPH r = 0.986, *p* < 0.01). When evaluating the dependence of antioxidant activity on the identified predominant phenolic *p*-coumaric acid, a medium intensity dependence was observed (FRAP r = 0.599; ABTS r = 0.650; DPPH r = 0.676, *p* < 0.05). The predominant flavonoid pinobanksin showed a stronger dependence on antioxidant activity compared to *p*-coumaric acid, and a strong correlation was observed between pinobanksin and antioxidant activity (DPPH r = 0.947; ABTS r = 0.966; FRAP r = 0.975, *p* < 0.01).

## 3. Discussion

Plant raw materials are rich in biologically active compounds, which can have antioxidant, anti-inflammatory, anticancer and antimicrobial effects [30,31]. Those qualities are especially prominent in phenolic compounds due to their chemical composition [32]. During our research it was determined that balsam poplar buds were rich in polyphenols, whose therapeutic value was widely described in different scientific studies [32,33,34]. In spite of being rich in polyphenols, the quality of raw material is greatly influenced by the extraction method and the chosen extractants [35]. One of the widely used solvents for raw material extraction in the pharmaceutical and food industries is ethanol [25,28]. For an optimal extraction of phenolic compounds from the raw material, the choice of solvent and such conditions as temperature and time are important [36,37]. Usually, when the extraction time and the temperature increase, the solubility of the material also increases, but the phenolic compounds in plants can break up when the extraction is too long or the temperature is too high and the undesirable process of oxidation occurs [36]. It is also important to choose a suitable solvent, because the solubility of active compounds differs [38,39].

For our research on the safe and environmentally friendly extraction of balsam poplar buds, we used such solvents as purified water and 70% ethanol (*v*/*v*). Those two extractants are important, as they affect the solubility of phenolic compounds differently. Isaeva et al. (2009) claimed that the major group of compounds of the ethanolic balsam poplar bud extract comprises neutral substances, 60% of which are accounted for by acyl glycerides and sterol ethers. The ethanolic extracts contain sesquiterpenoids and flavonoids, and these substances possess antimicrobial activity [40]. The results of our studies on the evaluation of the extracts’ quality showed that the amount of extracted phenolic compounds was higher when the ultrasound-assisted method, maceration and 70% ethanol (*v/v*) as solvent were used.

During our research we analysed the distribution of phenolic compounds in fresh and dried raw material extracts and determined that the smaller amount of phenolic compounds was found in extracts made from fresh plant material (25.56–184.11 mg CAE/g FW) compared to extracts made from dried raw material (48.19–225.86 mg CAE/g DW). When plant material is dried, the excess water is removed, which prevents the undesirable reproduction of microorganisms and increases the stability of the phenolic compounds in the raw material [41,42]. The drying process is critical, because it influences the antioxidant properties of the raw material, since the thermal decomposition and the action of oxidative enzymes are under way. Polyphenol oxidase is an oxidative enzyme which splits phenolic compounds, while peroxidase is associated with phytochemical splitting when hydrogen peroxide is present and plays an important role in splitting the phenolic compounds in the plant material, which are created by different drying methods [43]. The lower content of the active compounds in the extracts prepared from the fresh plant material may have been influenced by the fact that the fresh plant material usually has a higher moisture content [42].

The HPCL analysis allowed us to identify the predominant phenolic compounds. In the analysed balsam poplar bud extracts, the dominant phenolic acids were *p*-coumaric acid, cinnamic acid and flavonoids—pinobanksin and pinocembrin, the latter more expressed in the ethanolic extracts. It was stressed in the scientific literature that the chemical composition of poplar buds largely depends on the geographical and climate zones in which they grow. For example, Poblocka-Olech et al., after having conducted a thin-layer chromatography of Polish balsam poplar buds, identified different flavonoids (quercetin, galangin, chryzine, apigenin, pinocembrin and pinobanksin) [44], while in Brazilian poplar buds ferulic, caffeic and *p*-coumaric acids were found, along with campferol, chryzine, pinocembrin and pinobanksin [45]. Vardar-Ünlü et al.’s (2008) studies of the propolis extract samples showed similar GC-MS results with poplar bud extracts [46], that coincide with our initial investigation of the balsam poplar bud extracts’ chemical composition. The study also confirms that predominant phenolic compounds in poplar bud extracts coincide with Lithuanian propolis-predominant phenolic compounds [20]. Our studies have shown that the ethanolic extracts are dominated by *p*-coumaric, cinnamic and caffeic acids and pinocembrin and pinobanksin. During this research, a strong connection between the amount of phenolic compounds, the chosen solvent and the extraction methods was determined. In the investigated ethanolic extracts, a high amount of *p*-coumaric acid was found (5555.6–13,291.2 µg/g). This acid is widely analysed in the scientific literature, and the possibility to apply it as an anti-inflammatory, antioxidant and antitumor agent is being investigated [47,48]. For example, Jang et al. determined that the anticancer effect of *p*-coumaric acid can be associated with the modulation of RNA expression in SNU-16 gastric cancer cells [49].

The predominant flavonoids in the ethanolic balsam poplar bud extracts, such as pinobanksin and pinocembrin, have a good output, while water extracts have a small quantity of flavonoids. That can depend on the low solubility of the flavonoids in water [48]. The flavonoids pinocembrin and pinobanksin are often found in propolis samples from other countries [50], and they are important because of their strong antioxidant and anti-inflammatory effects. Soromou et al. carried out in vitro investigations and proved that pinocembrin suppressed anti-inflammatory cytokines in mouse macrophages and that endotoxin induced the acute pneumonia lung injury model, partly lessening the MAPK ir NF-κB levels of activation [51].

Another important compound identified and quantified in the poplar bud extracts studied was salicin. This phenolic compound is usually found in trees of the populus species, which contains various naturally-occurring aromatic compounds, such as salicylic acid, salicylic alcohol, salicin and other compounds [15,46]. In our research, aqueous extracts, infusions and decoctions had a statistically significant higher (*p* < 0.05) amount of salicin compared to the ethanolic extracts. Salicin is a water-soluble compound. Hot water increases its solubility [52], and it is possible to think that this had an influence on the larger amount of salicin content in infusions and decoctions compared to the ethanolic extracts.

The ability of the produced extracts to bind free radicals was investigated. The main applied methods of antioxidant activity were used: ABTS, DPPH, FRAP [53]. It should be noted that the antioxidant activity of the plant extracts, as determined by different antioxidant tests, may yield different results [54]. After the antioxidant activity using the ABTS, DPPH and FRAP methods, the results showed that all extracts had antioxidant activity, and the ability to bind free radicals increased depending on the extract output of phenolic compounds [55]. The results of our antioxidant study confirm the correlation between phenolic compounds and antioxidant activity. The ethanolic balsam poplar bud extracts had a statistically significant higher (*p* < 0.05) antioxidant activity (ABTS 680.76–753.28 µmol TE/g, DPPH 201.11–236.61 µmol TE/g, FRAP 713.22–751.63 µmol TE/g) in comparison with the aqueous balsam poplar bud extracts (ABTS 91.04–271.74 µmol TE/g, DPPH 31.16–124.07 µmol TE/g, FRAP 121.18–256.36 µmol TE/g). It is important to emphasize that the amount of phenolic compounds and their antioxidant activity are determined by the solubility of the active compounds in the extracts. Despite the results of in vitro antioxidant studies, the potential for in vivo antioxidant activity needs to be critically evaluated. In in vitro studies, the structure is better defined, since less metabolism takes place. Moreover, the concentration, a parameter that is directly linked to antioxidant activity, is more accurately controlled [56]. The reactions that occur in vivo are often too multifaceted and varied, so we cannot say that the same effect of antioxidant activity will be achieved both in vivo and in vitro. Extensive in vivo studies are needed to elucidate the therapeutic effects of poplar bud extracts.

The infectious pathogens that cause most infections in humans are the gram-positive bacteria *Staphylococcus aureus* and *Enterococcus faecalis* and the gram-negative bacteria *Pseudomonas aeruginosa* and *Escherichia coli* [57]. The antimicrobial properties of the extracts are extremely important for the application of balsamic poplar bud extracts for preventive and therapeutic purposes. The investigation data clearly showed that the investigated balsam poplar bud extracts had a stronger effect on gram-positive bacteria (*S. aureus* and *E. faecalis*) than on gram-negative bacteria (*E. coli* and *P. aeruginosa*). Nassima et al. investigated the antimicrobial activity of poplar buds. The results of their research showed the average activity of the extracts against gram-positive bacteria, including *S. aureus* ATCC 29213, *S. aureus* ATCC 6538, MRSA, *E. frendii* and *L. innocua*, and low activity against gram-negative bacteria, namely *E. coli* ATCC 25922, *E. coli* ATCC 8739 and *P. aeruginosa* [58]. Vardar-Ünlü et. al. concluded that poplar buds and propolis extracts demonstrated a comparable antimicrobial activity against gram-positive bacteria. The flavonoids and esters of phenolic acids are generally considered to be responsible for the antimicrobial activity of poplar buds and propolis extracts [46]. The results of our research showed that the ethanolic extracts had a higher output of phenolic compounds in comparison with aqueous extracts. The results of the research confirmed that the antimicrobial effect depends on the quantity of active compounds in the preparation.

Balsam poplar buds are a little-studied raw material, which is rich in biologically active compounds that are responsible for their antioxidant and antimicrobial activity. In our research we present knowledge about a new plant material, rich in biologically active compounds such as polyphenols, which is important to human health.

## 4. Materials and Methods

### 4.1. Materials

Solvents, standards and reagents of analytical grade were used. Purified deionized water was prepared with the Milli-Q^®^ water purification system (Millipore, Arlington, MA, USA). Rectified ethanol 96.3% (JSC “Vilniaus Degtine”, Vilnius, Lithuania) was used, along with the Folin-Ciocalteu reagent (Sigma-Aldrich, Buchs, Switzerland); 20% Chlorhexidine digluconate solution (Sigma-Aldrich, Steinheim, Germany) acetonitrile (>99.9%) (Sigma-Aldrich, Steinheim, Germany); reference standards: caffeic acid (≥98.0%), *p*-coumaric acid (≥98.0%), cinnamic acid (≥99.0%) (Sigma-Aldrich, Steinheim, Germany), chlorogenic acid (≥96.0%), vanillic acid (≥97.0%), pinobanksin (≥95.0%), pinocembrin (≥95.0%), galangin (≥95.0%) and salicin (≥99.0%) (Sigma-Aldrich, Buchs, Switzerland); sodium carbonate (≥99.0%) (Sigma-Aldrich, Saint-Quentin-Fallavier, France) and aluminum trichloride hexahydrate (≥99.0%) (Sigma-Aldrich, Steinheim, Germany). An ultrasonic bath (Bandelin electronic GmbH & Co.KG, Berlin, Germany) was used for the preparation of the extracts.

### 4.2. Balsam Poplar Bud Extracts

The balsam poplar buds were collected in northern Lithuania (Mazeikiai area) in March 2021 by the Jadvyga Balvočiūtė’s organic herb farm, and the collected fresh material was dried by the supplier. The solvents used in the extraction were purified water and 70% ethanol (*v*/*v*), with a ratio of raw material to extractant of 1:10 [28]. The extracts were obtained from fresh (No 02-03-2021) and dried (No 03-03-2021) plant raw material.

The balsam poplar buds were fully homogenized. To prepare the infusions and decoctions, the homogenized plant material was poured into a calculated amount of water. The infusions were prepared by pouring boiling water on the homogenized plant material, allowing it to steep for 15 min and cool down for 45 min. The decoctions were prepared by pouring cold water on the homogenized plant material, heating to a boil and allowing to simmer for 30 min and cool down for 15 min due to European Pharmacopoeia (2007) [27]. The prepared infusions and decoctions were filtered through ashless filter paper (ash content 0.007%, retention 8–12 µm, diameter 90 mm). The macerates were prepared by adding a selected amount of solvent to the homogenized plant material. The aqueous macerates were left to macerate for 5 days at 5 °C, well closed in the dark. The ethanol macerates were left to macerate for 7 days at room temperature, well closed in the dark. The ultrasonic extraction was performed in an ultrasonic bath for 60 min at 45 °C. All extracts were filtered and allowed to settle down overnight in a refrigerator (5 °C). The settled extracts were filtered to remove the ballast material and macromolecular compounds. Pure plant extracts were used for further research.

The prepared extracts were labelled according to the chosen raw material, the extraction method and the extractant. Prepared infusions from fresh raw material: A1I, and from dried material: B1I; produced decoctions from fresh material: A1D, and from dried material: B1D; aqueous macerates made from fresh material: A1M, and from dried material: B1M; 70% ethanolic (*v/v*) macerates from fresh materials: A2M, and from dried material: B2M; aqueous extracts prepared by ultrasound from fresh materials: A1U, and from dried material: B1U; 70% ethanolic (*v/v*) extracts prepared by ultrasound from fresh materials: A2U, and from dried material B2U.

### 4.3. Determination of Total Phenolic Compounds

The total phenolic compounds in balsam poplar bud extracts were evaluated spectrophotometrically with the Folin-Ciocalteu reagent, according to Singleton and Rossi (1965), with some modifications [59]. One hundred and fifity microliters of balsam poplar bud extracts were mixed with 750 µL of the Folin-Ciocalteu reagent, and after 3 min 600 µL of 7.5% sodium carbonate were added. The reaction mixtures were incubated for 2 h at room temperature, and the total amount of phenolic compounds were evaluated with a spectrophotometer (Agilent Technologies 8453 UV-Vis, Santa Clara, California, USA) at 760 nm. The results are expressed as mg of *p*-coumaric acid equivalent per 1 g of dry weight (mg CAE/g DW).

### 4.4. Determination of Falvonoids

The spectrophotometric determination of the flavonoid content was based on the Woisky and Sakatin (1998) method, with some modifications. To estimate the total flavonoid content, the balsam poplar bud extracts were diluted 5 times with 96% ethanol (*v/v*) in a 25 mL volumetric flask [60]. One mililiter of the diluted extract solutions, 10 mL of 96% ethanol (*v/v*), 2 mL of 10% AlCl_3_ and a few drops of 33% acetic acid were added to a 25 mL flask. The reaction mixtures were mixed and incubated for 20 min at room temperature, and then the reaction mixtures were diluted to the 25 mL mark with 96% ethanol (*v/v*). After incubation, the absorbance at 407 nm was measured. The results are expressed as mg of routine equivalent for 1 g (mg RE/g DW).

### 4.5. High Performance Liquid Chromatography (HPLC)

The identification of active compounds was performed by a high performance liquid chromatography. For the detection, a Waters 2695 chromatographic system with an ACE 5C18 chromatography column (250 × 4.6 mm) and a Waters 996 diode array detector was used. The resulting data were processed with the Empower 2 Chromatography Data Software. The eluent system consisted of 1% trifluoroacetic acid and 100% acetonitrile (the 1% trifluoroacetic acid—eluent A, 100% acetonitrile—eluent B), and the elution programme was used as follows: At 0–8 min from 5% to 15% B, at 8–30 min from 15% to 20% B, at 30–48 min from 20% to 40% B, at 48–58 min from 40% to 50% B, at 58–65 min from 50% to 50% B, at 65–66 min from 50% to 95% B, B at 66–70 min from 95% to 95%, at 70–71 min from 95% to 5% B. The mobile phase flow rate was 1 mL/min, the flow time 81 min, the injection volume 10 µL and the column temperature 25 °C. The compounds present in the extract samples were identified by the UV absorption (from 300 to 380 nm) and the retention time of the analytes and reference substances present [61]. Reference compounds: salicin (RT = 9.042), *p*-coumaric acid (RT = 19.669), caffeic acid (RT = 14.084), cinnamic acid (RT = 43.246), chlorogenic acid (RT = 11.538), vanillic acid (RT = 13.456), galangin (RT = 58.636), pinobanksin (RT = 47.158), pinocembrin (RT = 57.967). The results are presented as mean of three measurements and standard deviation.

### 4.6. Antioxidant Activity by the ABTS, DPPH and FRAP Methods

The antiradical activity of the extracts was determined by the ABTS and DPPH methods, with certain modifications by Yim et al. [62]. A stock solution of ABTS (0.0548 g ABTS, 50 mL purified water, 0.0095 g K_2_S_2_O_8_ (2 mmol/L)) was prepared. The ABTS working solution was prepared by diluting the stock solution with purified water until the absorption at a wavelength of 734 nm reached 0.8+/−0.03. The reaction mixture was prepared by mixing 3 µL of the balsam poplar bud extracts with 3000 µL of the ABTS working solution. The reaction mixtures were incubated for 30 min at room temperature. After incubation, the absorbance of the solutions was measured with a spectrophotometer (Agilent Technologies 8453 UV-Vis, Santa Clara, California, USA) at a wavelength of 734 nm.

The working solution of DPPH was prepared from the stock solution, diluting it with 96.3% ethanol until the working solution reached an absorbance 0.8 at 517 nm. Ten microliters of the prepared balsam poplar bud extracts mixed with 3000 µL of DPPH working solution. Samples incubated for 30 min. at room temperature. The absorbance measured with a spectrophotometer at a wavelength of 517 nm.

The FRAP-reducing activity was assessed based on the Benzie and Strain methodology [63], with some modifications. The working solution of FRAP was prepared from a 300 mmol/L sodium acetate buffer solution, a 10 mmol/L TPTZ solution and a 20 mmol/L FeCl_3_×6H_2_O aqueous solution (ratio 10:1:1). Ten microliters of balsam poplar bud extracts were mixed with 3000 µL of the FRAP working solution. All samples were incubated for 30 min at room temperature. The absorbance was measured with a spectrophotometer at a wavelength of 593 nm.

The calibration curve was established by using trolox standard solutions. The results of the balsam poplar buds’ antioxidant activity was expressed as the trolox equivalent per gram of tested raw material (µmol TE/g).

### 4.7. Antimicrobal Activity

The antibacterial properties of the balsam poplar bud extracts were evaluated using an agar diffusion method in vitro [64]. A Müller—Hinton agar (Mueller—Hinton agar Oxoid LTD (CM 0337), Basingstoke, Hampshire, England) was used as the culture medium. For the antimicrobial evaluation, gram-positive *Staphylococcus aureus* (Ref. ATCC 25923), *Staphylococcus aureus* (Wild), *Enterococcus faecalis* (Ref. ATCC 29212) and *Enterococcus faecalis* (Wild) and gram-negative *Escherichia coli* (Ref. ATCC 25922), *Escherichia coli* (Wild), *Pseudomonas aeruginosa* (Ref. ATCC 27853) and *Pseudomonas aeruginosa* (Wild) were used. The bacterial strains were spread on the surface of the medium, and six wells (7 mm) were made in each Petri dish and filled with 0.1 mL extract samples. The plates were incubated for 24 h at 37 °C. The antibacterial activity was evaluated after 24 h cultivation, measuring the diameter of the transparent areas around the wells. No transparent area was interpreted as negative antimicrobial activity. We used 0.5% chlorhexidine as a positive control (Sigma–Aldrich), and 70% ethanol (*v/v*) was used as a negative control.

### 4.8. Statistical Analysis

Different extraction conditions were tested using a two-way ANOVA test, and pairwise comparisons of extract concentrations were done using a Student’s t-test. To evaluate the data correlation, the Pearson correlation coefficient was determined (when 0.3 < |r| < 0.5, weak correlation; when 0.5 < |r| < 0.7, medium strength correlation; when 0.7 < |r| < 0.9, strong correlation; and when 0.9 < |r| ≤ 1, very strong correlation). The data were plotted and evaluated by IBM SPSS Statistics 27 (SPSS Inc., Chicago, IL, USA), Microsoft Office Excel 2016 (Microsoft, Redmond, WA, USA) and OriginPro^®^2021 (OriginLab, Northampton, MA, USA). The test results were obtained from three measurements, and the data obtained are expressed as mean and standard deviation (SD). The results were considered statistically significant when *p* < 0.05.

## 5. Conclusions

Balsam poplar buds have a narrow research spectrum. Preliminary studies of balsam poplar buds collected in Lithuania showed that this plant raw material is rich in polyphenols. A further HPLC analysis showed that the predominant phenolic acid in the raw material is *p*-coumaric acid, and the predominant flavonoids are pinocembrin and pinobanksin. The results of the analysis of the fresh raw material and the dried raw material showed that the dried raw material is a better source of polyphenols compared to the fresh raw material. The chosen solvent and the extraction method play an important role in the separation of polyphenols from the raw material. All test extracts had antioxidant activity, and the ethanol extracts had the best antioxidant activity. The ethanolic extracts of balsamic poplar buds showed a better antimicrobial activity against gram-positive bacteria. The role of polyphenols in human health is still widely studied. Based on the results of our research, the polyphenols found in balsamic poplar bud extracts offer much hope for further research on their application to human health.

## Figures and Tables

**Table 1:** Total phenolic compounds (mg CAE/g Fresh weight (FW) ± standard deviation (SD), dry weight (DW) ± SD, mean, *n* = 3) and total flavonoids (mg RE/g DW ± SD, FW ± SD, mean, *n* = 3) in 70% ethanolic (*v/v*) and aqueous balsam poplar bud extracts. Fresh and dried balsam poplar buds were used for extraction. The extracts made from fresh plant material are marked with the letter A, the extracts made from dried plant material are marked with the letter B. The extracts prepared by different methods are marked accordingly: I-infusions, D-decoctions, M-maceration, U-ultrasound. Different solvents were used for the extraction, which are denoted respectively: 1-purified water, 2-70% ethanol (*v/v*). The data are presented as mean and standard deviations (SD).

Plant Material	Solvent	Extraction Method	Sample Marking	Total Phenolic Compounds mg CAE/g FW	Total Flavonoids mg RE/g FW
A group of extracts prepared from fresh balsam poplar buds	Purified water	Infusion (I)	A1I	25.56 ± 0.46	2.85 ± 0.35
		Decoction (D)	A1D	43.05 ± 1.67	3.35 ± 0.18
		Maceration (M)	A1M	77.91 ± 3.09 ^a,b^	7.55 ± 0.13 ^d^
		Ultrasound (U)	A1U	84.49 ± 1.38 ^b^	8.59 ± 0.29
	70% ethanol (*v/v*)	Maceration (M)	A2M	162.61 ± 7.91	45.58 ± 2.54 ^e^
		Ultrasound (U)	A2U	184.11 ± 7.97	51.64 ± 2.63
B group of extracts prepared from dried balsam poplar buds	Purified water	Infusion (I)	B1I	48.19 ± 2.46	4.62 ± 0.2+40
		Decoction (D)	B1D	72.54 ± 1.82 ^a^	7.01 ± 0.70 ^d^
		Maceration (M)	B1M	97.38 ± 7.35	9.84 ± 0.71 ^f^
		Ultrasound (U)	B1U	114.43 ± 3.36	9.92 ± 0.58 ^f^
	70% ethanol (*v/v*)	Maceration (M)	B2M	212.73 ± 8.11 ^c^	39.33 ± 2.05
		Ultrasound (U)	B2U	225.86 ± 13.17 ^c^	45.26 ± 2.29 ^e^

Pairwise comparisons using a Student’s *t*-test were performed for each extraction method, comparing pairs for phenol and flavonoid samples separately. The shared letters in superscript indicate that there was no statistically significant difference between the concentrations (*p* > 0.05).

**Table 2:** HPLC analysis of 70% ethanolic (*v/v*) and aqueous balsam poplar bud extracts (µg/g Fresh weight (FW) ± standard deviation (SD), dry weight (DW) ± SD, mean, *n* = 3). Fresh and dried balsam poplar buds were used for extraction. The extracts made from fresh plant material are marked with the letter A, the extracts made from dried plant material are marked with the letter B. The extracts prepared by different methods are marked accordingly: I-infusions, D-decoctions, M-maceration, U-ultrasound. Different solvents were used for the extraction, which are denoted respectively: 1-purified water, 2-70% ethanol (*v/v*). Much higher concentrations of *p*-coumaric acid (F(1) = 1743.50, *p* < 0.001) and cinnamic acid (F(1) = 1682.93, *p* < 0.001) were found in the dried poplar buds compared to the fresh poplar buds.

Plant Material	A Group of Extracts Prepared from Fresh Balsam Poplar Buds µg/g (FW ± SD)					
Solvent	Purified Water				70% Ethanol (*v/v*)	
Extraction Method	Infusion (I)	Decoction (D)	Maceration (M)	Ultrasound (U)	Maceration (M)	Ultrasound (U)
Sample Marking	A1I	A1D	A1M	A1U	A2M	A2U
Salicin	868.4 ± 42.3	1190.7 ± 60.9	410.1 ± 9.1	727.6 ± 33.3	318.4 ± 13.6	243.2 ± 12.0
Chlorogenic acid	-	-	-	192.3 ± 6.9	102.2 ± 4.8	307.4 ± 13.8
Vanilic acid	2.4 ± 0.1	2.6 ± 0.1	47.3 ± 2.3	0.8 ± 0.01	7.62 ± 0.4	-
Caffeic acid	124.9 ± 5.7	190.4 ± 4.2	793.4 ± 45.8	593.7 ± 32.5	542.2 ± 23.3	392.3 ± 19.8
*P*-coumaric acid	496.9 ± 16.3 ^a^	907.8 ± 36.7 ^b^	621.3 ± 31.9 ^a,b^	2032.9 ± 81.6	2730.8 ± 171.4	5555.6 ± 215.9
Cinnamic acid	32.9 ± 1.6 ^a^	150.6 ± 5.8 ^b^	96.4 ± 4.2 ^a,b^	491.9 ± 22.5^c^	423.2 ± 17.6 ^c^	2441.0 ± 156.1
Pinobanksin	34.9 ± 2.1	41.9 ± 2.4	41.0 ± 2.0	41.8 ± 1.6	1393.9 ± 77.8	1751.8 ± 120.7
Pinocembrin	-	-	-	-	1916.5 ± 108.8	1556.5 ± 46.4
Galangin	-	-	-	-	848.2 ± 55.4	1492.6 ± 24.7
Total identified phenolic acids	657.1	1251.4	1558.4	3311.6	3806	8696.3
Total identified flavonoids	34.9	41.9	41	41.8	4158.6	4800.9
Total amount of identified active compounds	1560.4	2484	2009.5	4081	8283	13,740.4
**Plant Material**	**B Group of Extracts Prepared from Dried Balsam Poplar Buds µg/g (DW ± SD)**					
**Solvent**	**Purified Water**				**70% Ethanol (*v/v*)**	
**Extraction Method**	**Infusion (I)**	**Decoction (D)**	**Maceration (M)**	**Ultrasound (U)**	**Maceration (M)**	**Ultrasound (U)**
**Sample Marking**	**B1I**	**B1D**	**B1M**	**B1U**	**B2M**	**B2U**
Salicin	819.0 ± 30.2 ^a^	617.8 ± 24.5	455.0 ± 12.2	767.6 ± 36.5 ^a^	224.7 ± 13.4 ^b^	215.3 ± 12.1 ^b^
Chlorogenic acid	223.7 ± 11.9	346.1 ± 18.1	283.4 ± 17.1	280.1 ± 14.8	315.9 ± 17.9	310.3 ± 20.4
Vanilic acid	0.45 ± 0.03	0.61 ± 0.02	6.9 ± 0.4	-	0.6 ± 0.03	-
Caffeic acid	288.4 ± 16.4	481.9 ± 32.9	1159.5 ± 90.9	1179.9 ± 84.2	665.9 ± 35.6	735.1 ± 41.6
*P*-coumaric acid	4762.7 ± 299.8 ^a^	7869.8 ± 294.4	5974.9 ± 326.8	3896.6 ± 166.3 ^a^	10,165.0 ± 319.3	13,291.2 ± 224.6
Cinnamic acid	2536.5 ± 154.3	3720.1 ± 165.5	872.2 ± 46.9	1815.2 ± 101.4	8529.2 ± 155.7	11,788.5 ± 384.0
Pinobanksin	67.8 ± 3.8	110.9 ± 6.5	35.7 ± 1.8	16.7 ± 0.8	1328.1 ± 128.5	1775.5 ± 106.5
Pinocembrin	-	-	0.8 ± 0.06	2.4 ± 0.2	1014.6 ± 67.5	810.6 ± 36.5
Galangin	-	-	-	3.5 ± 0.2	745.7 ± 35.9	1431.4 ± 100.7
Total identified phenolic acids	7811.8	12,418.5	8296.9	7171.8	19,676.6	26,125.1
Total identified flavonoids	67.8	110.9	36.5	22.6	3088.4	4017.5
Total amount of identified active compounds	8698.6	13,147.2	8788.4	7962	22,989.7	30,357.9

Pairwise comparisons using a Student’s *t*-test were performed for each compound, comparing pairs for the fresh and dried samples separately. The shared letters in superscript indicate that there was no statistically significant difference between the concentrations (*p* > 0.05).

**Table 3:** Antimicrobal activity of the extracts of balsam poplar buds (Ømm ± SD). The antimicrobial activity of the dried samples was different from that of the raw samples (F(3) = 88.49, *p* < 0.001), and the samples were more active against gram-positive strains compared to gram-negative strains (F(4) = 150.06, *p* < 0.001). Fresh and dried balsam poplar buds were used for extraction. The extracts made from fresh plant material are marked with the letter A, the extracts made from dried plant material are marked with the letter B. The extracts prepared by different methods are marked accordingly: I-infusions, D-decoctions, M-maceration, U-ultrasound. Different solvents were used for the extraction, which are denoted respectively: 1-purified water, 2-70% ethanol (*v/v*). The data are presented as mean and standard deviations (SD). Ni denotes no inhibition.

Bacterial Strain	*S. aureus* Ref. ATCC 25923	*S. aureus* Wild	*E. faecalis* Ref. ATCC 29212	*E. faecalis* Wild	*E. coli* Ref. ATCC 25922	*E. coli* Wild	*P. aeruginosa* Ref. ATCC 27853	*P. aeruginosa* Wild
Zone of inhibition	Ø mm	Ø mm	Ø mm	Ø mm	Ø mm	Ø mm	Ø mm	Ø mm
A1I	NI	NI	NI	NI	NI	NI	NI	NI
A1D	NI	NI	NI	NI	NI	NI	NI	NI
B1I	NI	NI	NI	NI	NI	NI	NI	NI
B1D	<5	<5	NI	NI	NI	NI	NI	NI
A1M	<5	<5	NI	NI	NI	NI	NI	NI
B1M	5.3 ± 0.5	<5	<5	<5	NI	NI	NI	NI
A1U	<5	<5	NI	NI	NI	NI	NI	NI
B1U	5.3 ± 0.5	5.3 ± 0.5	<5	<5	NI	NI	NI	NI
A2M	18.7 ± 0.6 ^a,b^	17.3 ± 1.0 ^a,c^	16.7 ± 1.0 ^b,c^	13.3 ± 0.5	8.3 ± 0.5	NI	NI	NI
B2M	26.7 ± 0.6 ^a^	25.3 ± 0.5 ^a^	19.3 ± 0.5 ^b^	17.3 ± 0.5 ^b^	11.3 ± 0.5	NI	NI	NI
A2U	17.3 ± 1.0 ^a,c^	15.7 ± 1.0 ^a,d^	14.3 ± 0.5 ^b,c,d^	12.7 ± 0.6 ^b^	7.7 ± 0.6	NI	NI	NI
B2U	27.6 ± 1.0 ^a^	25.7 ± 1.0 ^a^	20.0 ± 1.6 ^b^	18.3 ± 1.0 ^b^	11.7 ± 0.6	NI	NI	NI
70% Ethanol (*v/v*)	6.3 ± 1.0	6.0 ± 0.0	5.6 ± 0.5	5.3 ± 0.5	<5	NI	NI	NI
Positive control 0.5% chlorhexidine	22.3 ± 1.0	20.8 ± 0.6	24.3 ± 0.5	17.6 ± 0.5	22.3 ± 0.5	18.7 ± 0.6	20.3 ± 0.5	19.6 ± 0.5

Pairwise comparisons using a Student’s *t*-test were performed for each pair of bacterial strains for each extraction method separately. The shared letters in superscript indicate that there was no statistically significant difference between the concentrations (*p* > 0.05).

**Table 4:** Correlation graph of the total phenolic compounds, antioxidant activity (ABTS, DPPH, FRAP), *p*-coumaric acid and pinobanksin in 70% ethanolic (*v/v*) and aqueous balsam poplar bud extracts by a Pearson correlation.

Correlations						
	**Total Phenolic Compounds**	**ABTS**	**DPPH**	FRAP	*P*-Coumaric Acid	Pinobanksin
Total Phenolic Compounds	--					
ABTS	0.974 **	--				
DPPH	0.986 **	0.996 **	--			
FRAP	0.955 **	0.994 **	0.985 **	--		
*P*-Coumaric Acid	0.728 **	0.650 *	0.676 *	0.599 *	--	
Pinobanksin	0.907 **	0.966 **	0.947 **	0.975 **	0.602 *	--

**. Correlation is significant at the 0.01 level; * Correlation is significant at the 0.05 level.

## Data Availability

Data available in a publicly accessible repository.
